# Decreased expression of circulating Aire and increased Tfh/Tfr cells in myasthenia gravis patients

**DOI:** 10.1042/BSR20180096

**Published:** 2018-11-09

**Authors:** Sijia Zhao, Jiaqi Ding, Shengyuan Wang, Chuan Li, Peng Guo, Min Zhang, Zhuyi Li

**Affiliations:** 1 Department of Neurology, Tangdu Hospital, Fourth Military Medical University, Xi’an, Shaanxi 710038, China; 2 Department of Hyperbaric Oxygen, Navy General Hospital of Chinese People’s Liberation Army, Beijing 100048, China

**Keywords:** circulating Autoimmune regulator, circulating Follicular helper T cell, circulating Follicular regulatory T cell, Myasthenia gravis

## Abstract

Myasthenia gravis (MG) is a rare prototypical autoimmune disorder caused by antibodies (Ab) against postsynaptic membrane proteins. Most reports have investigated the role of autoimmune regulator gene (Aire) in thymic tissue in machianism of MG initiation. So far, the expression of Aire in human peripheral blood cells (we call it circulating Aire expression in the following passage) has not been reported. Herein, we explore the expression of Aire in peripharal blood, circulating T-follicular helper (cTfh) and T-follicular regulatory (cTfr) cells in MG patients. In our research, we found that the acetylcholine receptor (AChR) Ab level is higher in generalized MG (GMG) than that in ocular MG (OMG). Compared with the control group (CG), lower expression of Aire was found in MG patients, especially in GMG. The ratio of Tfh/Tfr was higher in GMG patients, and then in the OMG patients, and lowest in CG. All these differences above were statistically significant. Negative relation was discovered between expression of Aire in circulating blood and ratio of Tfh/Tfr, so did it exist between Aire expression and the severity of MG. Meanwhile, positive relation was discovered between ratio of Tfh/Tfr and the severity of MG. However, no significant relation was manifested in our study between the subset age of MG and Aire level. Overall, these findings imply circulating Aire might play a role in the imbalance of cTfh and cTfr cells and participate in the pathogenesis of MG.

## Introduction

Myasthenia gravis (MG) is an autoimmune disease triggered by antibodies against the acetylcholine receptor (AChR) of skeletal muscle at the neuromuscular junction (NMJ). Eighty to ninety percent of MG patients initialing with extraocular muscle weakness, which is called ocular MG (OMG), will develop to generalized MG (GMG). GMG manifesting as skeletal or bulbar muscle weakness within 2 or 3 years [1]. Thus, to clarify the different pathogenic mechanism between OMG and GMG is the hinge to restrain disease progression.

It is generally accepted that thymus is the origination part of MG. But MG recurrences after thymectomy lead us to doubt if there is something outside thymus causing MG recrudescence. Till now, the pathogenesis of MG outside thymus remains unclear.

Autoimmune regulator gene (Aire) is first reported as a transcription factor expressed by medullary thymic epithelial cell that promotes the ectopic expression of tissue-restricted antigen [2]. The induction of genes involved in antigen processing and presentation [3], and in the production of chemokines that impacts the density of dendritic cells in the medulla [4]. Mutation of thymic Aire gene is responsible for the development of autoimmune disease [5]. It was found that Aire is 8.5-fold more expressed in non-neoplastic thymi than in thymomas of MG patients A [6]. Polymorphism in Aire gene, especially the rs3761389, may associate with the susceptibility of MG [7]. Meanwhile, the abnormal expression of Aire in thymi could lead to increased autoreactive T cells, which induced impaired immune tolerance, and consequently resulted in autoimmune diseases [8].

A subset of CD4^+^ T cells, named follicular helper T (Tfh) cells, is specialized for promoting B cells in germinal centers (GCs) [9]. Formation of GCs in thymi is the primary thymi characteristic in MG patients [10,11], which up-regulated activated B cells [12]. Tfh cells can promote B cells to form plasma cells, generating a large number of high affinity antibodies [13]. Follicular regulatory T (T follicular regulatory, Tfr) cells play a role with dual characteristics of Tfh and Treg cells [14] by powerfully inhibiting self-reactive cells. Recent studies have shown that the deficiency of Tfh/Tfr cell balance may influence the severity of MG [15].

Most reports have investigated the role of Aire and Aire gene in thymic tissue in machianism of MG initiation. So far, the expression of Aire in human peripheral blood cells (we call it circulating Aire expression in the following passage) has not been reported. Till now, there was no conclusion on whether Aire is related with Tfh/Tfr cell balance in MG patients. The present study is to compare the expression of Aire, ratio of Tfh/Tfr cells in MG patients, and then to explore the relationship between the expression and the severity of MG accordingly.

## Methods

### Samples

Twenty-two MG patients in our institution (Neurology Department, Tangdu Hospital, Fourth Military Medical University) were enrolled from December 2016 to April 2017, including 12 GMG patients (six females and six males, average age: 47.75 years) recruited as GMG group and 10 OMG patients (two females and eight males, average age: 42.5 years) recruited as OMG group. Meanwhile, 10 healthy subjects (four females and six males, average age: 40.7 years) were selected from Center of Health Examination in the same hospital as control group (CG). The research was approved by Ethics Committee of the Faculty of Tangdu Hospital. All patients provided informed,written consent to participate in the study.

### Patients

OMG group inclusion criteria were: (i) had a diagnosis of OMG according to clinical manifestation: verified by an examination of muscular fatigability in any ocular muscles and other muscle strength normal; (ii) AChR antibodies are positive and a decreased response to repetitive motor nerve stimulation; (iii) classified in class according to the Myasthenia Gravis Foundation of America (MGFA) clinical classification; (iv) patients without any immunosuppressive therapy including prednisone or other immunosuppressive agents.

GMG group inclusion criteria were: (i) had a diagnosis of GMG according to clinical manifestation: verified by an examination of muscular fatigability in limb, axial, oropharyngeal, or respiratory muscles and any ocular muscle; (ii) AChR antibodies are positive and a decreased response to repetitive motor nerve stimulation; (iii) classified in classes Ⅱ– according to the MGFA clinical classification; (iv) patients without any immunosuppressive therapy including prednisone or other immunosuppressive agents.

Exclusion criteria were: (i) seronegative and muscle-specific kinase (MuSK) positive patients were excluded, as they may have different initiation of pathogenesis from AChR-positive patients; (ii) patients with other comorbidities and immune diseases; (iii) received immunosuppressive therapy within the past 3 months; (iv) exhibited an acute inflammation within the preceding 4 weeks; (v) received a thymectomy prior to the study.

Eligible samples for CG included: (i) health investigated indexes were normal; (ii) without any autoimmune disease; (iii) health people did not use glucocorticoids or any immunosuppressant therapy in 1 year. Clinical characteristics of MG patients and CG are summarized in Table 1.

**Table 1:** Clinical characteristics of MG patients and CG

Number	Group	Gender	Age (years)	MGFA	QMGs	AChR-Ab (nmol/l)
1	GMG	M	61	IVb	27	>8
2	GMG	M	54	IVb	17	>8
3	GMG	F	34	IVb	16	>8
4	GMG	M	34	IIIb	12	>8
5	GMG	M	52	IIIb	15	>8
6	GMG	M	51	IIIb	24	5
7	GMG	F	47	IIIa	21	5
8	GMG	F	37	IIIa	11	>8
9	GMG	M	28	IIb	13	>8
10	GMG	F	58	IIb	15	1.42
11	GMG	F	51	IIb	5	>8
12	GMG	F	66	IIb	8	>8
13	OMG	M	29	I	12	5
14	OMG	M	61	I	3	1.4
15	OMG	M	21	I	11	1.5
16	OMG	M	58	I	6	3.51
17	OMG	M	63	I	3	1.3
18	OMG	M	57	I	9	7.8
19	OMG	M	45	I	6	>8
20	OMG	M	21	I	3	1.3
21	OMG	F	50	I	6	>8
22	OMG	F	20	I	10	1.36
23	CG	M	46	N/A	N/A	N/A
24	CG	M	24	N/A	N/A	N/A
25	CG	M	64	N/A	N/A	N/A
26	CG	M	56	N/A	N/A	N/A
27	CG	M	52	N/A	N/A	N/A
28	CG	M	22	N/A	N/A	N/A
29	CG	F	43	N/A	N/A	N/A
30	CG	F	50	N/A	N/A	N/A
31	CG	F	25	N/A	N/A	N/A
32	CG	F	25	N/A	N/A	N/A

Abbreviations: AChR-Ab, titer of AChR antibody; F, female; M, male; N/A, not available; QMG, quantitative MG score.

### Data and sample collection

The basic clinical information of patients was collected including name, gender, age, clinical manifestations, physical examinations, and onset age. The clinical type of MG was divided by MGFA. The clinical severity of MG was accessed by quantitative MG scoring system (QMGs). MGFA and QMGs were collected by two skilled physicians until they reached a consensus.

The blood samples of MG patients were collected for examination of circulating Aire expression, CD4+CXCR5+Foxp3− (Tfh cells) and CD4+CXCR5+Foxp3+ (Tfr cells) before treatment.

All the MG patients had detectable anti-AChR antibodies by RIA testing at the time of entry into the study (Athena Diagnostics, Worcester, MA, U.S.A.)

### Sample processing

Six milliliters of peripheral blood sample was collected from the included MG and CG individuals and put into a heparinized tube. The peripheral blood mononuclear cells were isolated via density-gradient centrifugation with the lymphocyte separation medium (LTS1077, TBD Sciences, CHN) at room temperature (RT). And cells were washed twice with PBS (pH 7.2) within 3 h.

### Flow cytometric

The cells were resuspended up to 10^6^ nucleated cells and divided into tubes A and B. In tube A, the cells were incubated with 5 μl CD4-FITC (317407, Biolegend, U.S.A.) and CD185-PE (CXCR5) (356903, BioLegend, U.S.A.) in the dark at RT for 30 min. Then, the tissues were washed twice with PBS for intercellular staining.

Cells in tubes A and B were incubated in 1 ml cold, freshly prepared fixation/permeabilization solution (130-093-142, MACS, GER) in the dark at 4°C for 30 min. After washing twice with permeabilization buffer, the cells were separately stained with Foxp3- Alexa Flour (320013, BioLegend, U.S.A.) and 10 μl Anti-human Aire-APC (130-093-142, MACS, GER) and next incubated for 30 min at RT. The stained cells were detected by flow cytometry immediately.

To guarantee the accuracy of the result, isotype controls were used to determine the gating parameters. FACS Calibur instrument (Becton Dickinson, U.S.A.) was used to conduct flow cytometry.

### Statistical analysis

The expression of circulating Aire, Tfh, and Tfr cells was analyzed by one-way ANOVA to determine whether there were statistically significant changes amongst GMG, OMG, and CG groups. Linear regression analysis was done to determine the correlations between Aire expression, Tfh/Tfr ratio, and clinical severity of GMG by QMGS, respectively. All tests were two-tailed, and statistical significance was set at *P*<0.05. All statistical analysis was performed with SPSS13.0 for windows software (SPSS Inc., U.S.A.).

## Result

**Figure 1:**
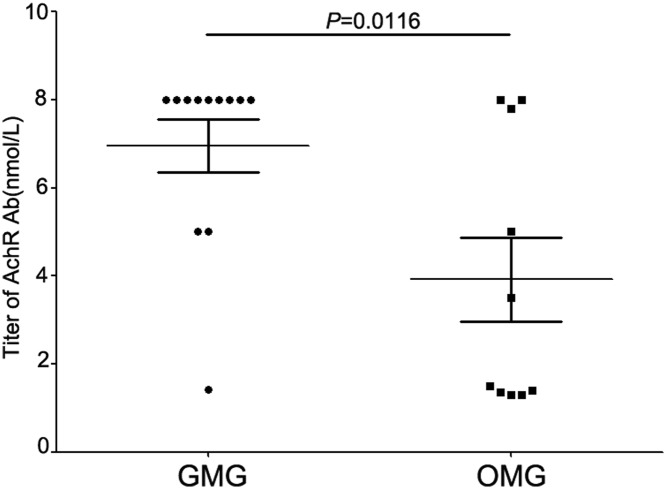
Higher titer of AChR Ab in GMG is statistically higher than that in OMG patients Peripheral blood was obtained from 22 MG patients. We detected the titer of serum AChR Ab in patients with OMG or GMG by RIA. Of all included patients, the titer of AChR Ab in GMG is statistically higher than that in OMG patients (*P*=0.004).

Twenty-two MG patients and 10 age-matched CG were enrolled in the present study, and MG patients included 12 GMG and 10 OMG. All the data were shown in Table 1. Before research, we detected the titer of serum AChR antibody (Ab) in patients with OMG or GMG by RIA. Of all included patients, the titer of AChR Ab in GMG is statistically higher than that in OMG patients (*P*=0.004) (Figure 1).

### Lower Aire, cTfr cells, and higher cTfh cells in GMG than in OMG and CG

**Figure 2:**
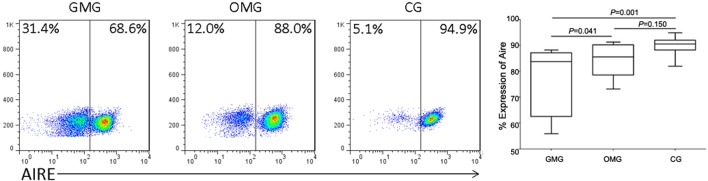
Lower Aire in GMG than in OMG and CG The comparison of frequencies of Aire+ cells in HC and MG patients. Compared with OMG and CG, lower expression of Aire was found in GMG (*P*=0.041, *P*=0.001, respectively). And difference between OMG and CG did not reach significantly (*P*=0.159).

Lowest expression of Aire was found by flow cytometry in GMG than that in OMG and CG (*P*=0.041, *P*=0.001, respectively), while it reached no statistical significance between OMG group and CG (*P*=0.159) (Figure 2).

**Figure 3:**
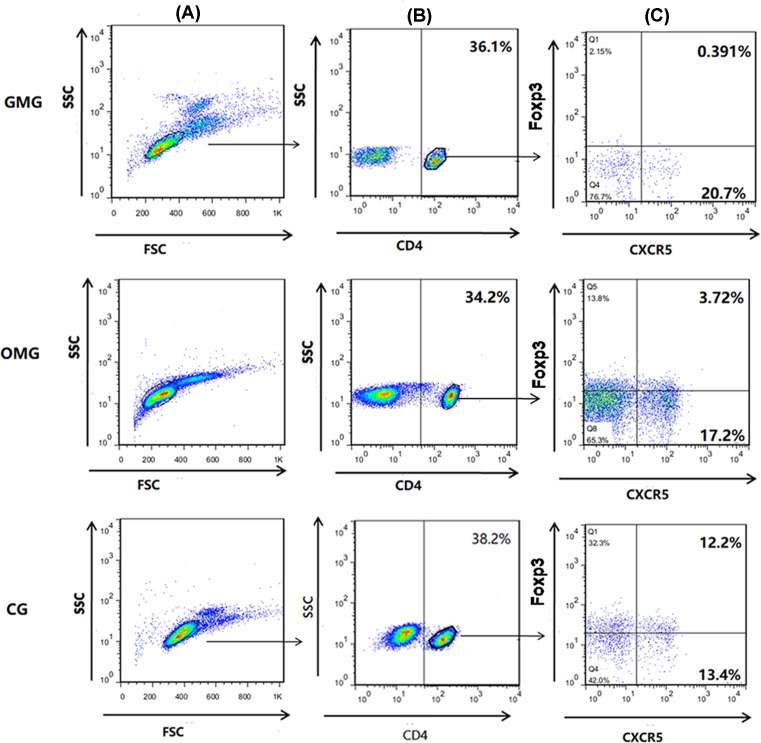
Lower cTfr cells and higher cTfh cells in GMG than in OMG and CG We compared the Tfr cells and Tfh cells expression in GMG, OMG, and CG. (**A**) Isolated lymphocytes from GMG, OMG, and CG. (**B**) Gated for the expression of CD4 cells from the lymphocytes. (**C**) Values in the upper right quadrant correspond to the frequency of CD4+CXCR5+Foxp3+ T cells (cTfr cells). And the bottom right quadrant corresponds to the frequency of CD4+CXCR5+Foxp3− T cells (cTfh cells).

**Figure 4:**
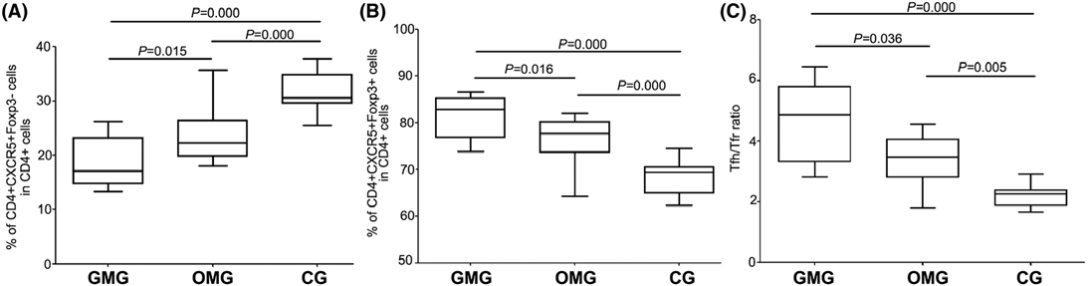
Higher ratio of cTfh/cTfr in GMG than in OMG and CG Higher cTfh cells were found in GMG group than that in OMG and CG, and this difference reached significantly (*P*=0.016, *P*=0.001, respectively). And difference between OMG and CG also reached significantly (*P*=0.000) (**A**). Lower cTfr cells were found in GMG group than that in OMG and CG, and this difference reached significantly (*P*=0.015, *P*=0.001, respectively). And difference between OMG and CG also reached significantly (*P*=0.000). (**B**) Higher ratio of cTfh/cTfr was found in GMG group than that in OMG and CG, and this difference reached significantly (*P*=0.036, *P*=0.000, respectively). And difference between OMG and CG also reached significantly (*P*=0.005) (**C**).

The result of flow cytometry indicated the lowest expression of cTfr cells in GMG than that in OMG and CG (*P*=0.015, *P*=0.000, respectively), and it was also found to be statistically significant between OMG group and CG (*P*=0.000) (Figures 3 and 4A). Flow cytometry results indicated the highest expression of cTfh cells in GMG than that in OMG and CG (*P*=0.016, *P*=0.000, respectively), and it was also found to be statistically significant between OMG group and CG (*P*=0.000) (Figures 3 and 4B).

cTfh and cTfr cells are all CD4+CXCR5+ T cells and the difference between these two groups was the expression of Foxp3. Both cTfh and cTfr are closely related and affect each other. So, it is necessary to explore the imbalance of circulating CD4+CXCR5+ T cells. Herein, we used the ratio of Tfh/Tfr to present this imbalance.

### Different expression of AChR-antibody in GMG and OMG patients

In our research, we found that the ratio of cTfh/cTfr was highest GMG, then in OMG, and the lowest in CG. The difference reached significantly in GMG, compared with OMG and CG. (*P*=0.036, *P*=0.000, respectively) (Figure 4C). and it was also found to be statistically significant between OMG group and CG (*P*=0.005).

### Aire expression is negative correlated with Tfh/Tfr ratio and neither Aire, nor Tfh/Tfr shows significant relation with subset age of MG

**Figure 5:**
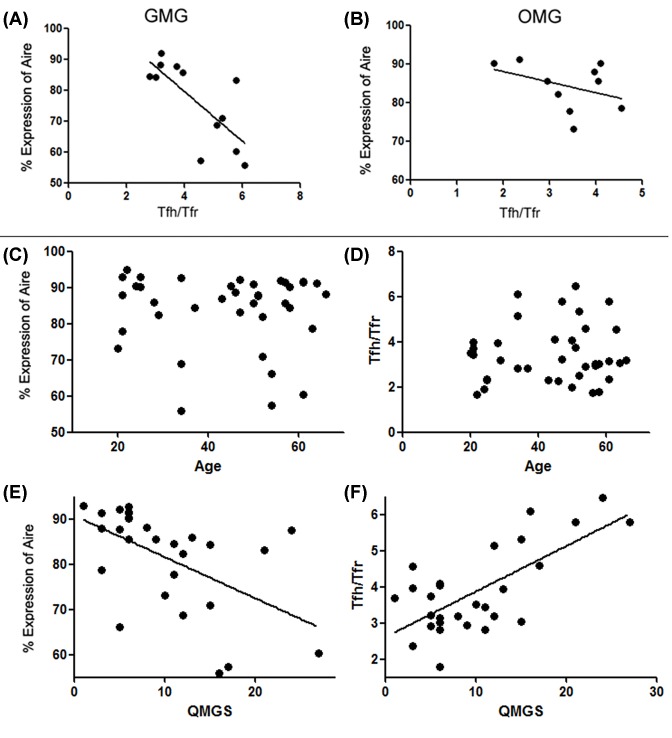
Negative correlation between circulating Aire level and Tfh/Tfr ratio in GMG Little correlation between circulating Aire level, Tfh/Tfr ratio, and subset age of MG. Negative correlation between circulating Aire level and QMGs, positive correlation between cTfh/cTfr ratio and QMGs. Negative correlation between circulating Aire level and Tfh/Tfr ratio in GMG was discoved in GMG (*F* = 25.031; *P*=0.000, *r* = −0.674) (**A**), and in OMG, the correlation did not reach significantly (**B**). Little correlation was found neither between circulating Aire level and subset age of MG (**C**), nor between Tfh/Tfr ratio and subset age of MG (**D**). Negative correlation between circulating Aire level and QMGs (*F* = 21.952; *P*=0.000, *r* = −0.487) (**E**). Similarly, a positive correlation was found between cTfh/cTfr ratio and QMGs (*F* = 58.493; *P*=0.000, *r* = 0.813) (**F**).

Regression analysis showed a negative correlation between circulating Aire level and Tfh/Tfr ratio in GMG (*F* = 25.031; *P*=0.000, *r* = −0.674) (Figure 5A). And in OMG, the correlation did not reach significantly (Figure 5B). However, little correlation was found between circulating Aire level and subset age of MG (Figure 5C) as well as the correlation between circulating Tfh/Tfr and subset age of MG (Figure 5D).

### Aire expression and cTfh/cTfr ratio were all related to the severity of MG status measured by QMG scores

Regression analysis showed a positive correlation between circulating Aire level and the severity of MG (*F* = 21.952; *P*=0.000, *r* = −0.487) (Figure 5E). Similarly, a positive correlation was found between cTfh/cTfr ratio and MG disease severity (*F* = 58.493; *P*=0.000, *r* = 0.813) (Figure 5F).

## Discussion

As is known, dysregulation of Tfh and Tfr cells is associated with pathogenesis of autoimmune diseases, such as MG, systemic lupus erythematosus, rheumatoid arthritis, and plays an indispensable role in adaptive immunity through accommodation of B cells, immunoglobulin, and plasma cells [16–18]. Recent study has revealed that compared with healthy controls; there is a significantly decreased frequency of Tfr-like cells and an increased frequency of Tfh-like cells in the peripheral blood of MG patients. And also at the same time, Wen et al. [15] also found a negative relationship between the severity of MG and the Tfr/Tfh ratio. Our study also confirms the dysfunction of Tfh/Tfr cells in MG patients and this dysfunction is positive related with the severity of MG. But the mechanism of this dysfunction is still not fully understood.

Aire was first reported to be associated with MG in 2002. It was recognized that Aire-deficient thymic medullar epithelial cells exhibited a decrease of expression of peripheral antigens [6]. Then in 2010, observation was followed which unveiled that the mutation of epithelial expression Aire gene in B2 thymoma induces the absence of myoid cells, thus resulting in the loss of Foxp3+ T cells. And the lost Foxp3+ T cells perhaps related with the impairing negative selection in thymoma [19]. All this autoimmune procedure might induce the abnormal expression of AChR, which involved in the pathogenesis of MG. According to a report, the young Aire(−/−) mice tends to have increased numbers of Treg cells in their spleens and more susceptibility of autoimmune disease, such as MG [20]. Moreover, less expression of Aire was found in normal female mice thymus than that in males. However, reducing expression of thymic Aire in male mice has more tendencies to autoimmune disease, such as experimental autoimmue thyroiditis (EAT) [21].

To our knowledge, there is little report on Aire in peripheral blood but large on thymus. A possible involvement of Aire in peripheral is that Aire-expressing dendritic cells inhibited TCR signaling pathways and caused the depletion of CD4+IFN-γ+ autoreactive T cells in auto-immune disease mice models [22]. In other studies, it is observed that *in vitro*, overexpressing-Aire macrophages promote the number of CD4+Foxp3+ Treg cells. We notice there may be a distinct relationship between thymic and peripheral blood. Lacking Aire expression research in human peripheral blood, we wonder if there exists same tendency of Aire expression, and also relationship of Aire and Tfh/Tfr cells in peripheral blood.

We found a deficient expression of Aire, Tfr cells in peripheral blood, and also a higher expression of Tfh cells than healthy controls. Following statistics showed a negative relationship between Aire expression in peripheral blood and the severity of MG. Meanwhile, higher Tfh/Tfr cells ratio related to the severity of MG was illustrated. At the same time, negative relationship between lower expression of Aire and higher Tfh/Tfr cells ratio was noticed. Now, consider above all these studies: deficit of expression of AIRE gene is probably related to the absence of Foxp3+ T cells. The absence of Foxp3 leads to decrease in Tfr cells. Then, the disporpotion of Tfh/Tfr cells might induce an abnormal autoimmune disease, such as MG, and this influences the severity of disease.

Till now, accumulated Aire-associated reports suggested that AIRE gene knockout mice are more likely to induce profile of specific autoimmune disease, and correlated with age [20]. It is illustrated in the report that the older the more likely to induce the onset of MG. But in our research, AIRE expression and Tfh/tfr were not related to gender and age, which is different from the reported results. We attribute this difference to three aspects. First, there perhaps exist distinct pathways in thymus and peripheral blood, therefore the blood results may not fully consistent with the thymus results. Then, experimental autoimmune MG model cannot fully imitate clinical MG. For example, experiments cannot simulate mice with thymoma and MG at the same model. Last, the relationship between susceptibility and age in mice might be attributed to degenerative immunity in aged mice.

Taken together, we have demonstrated for the first time the differential expressions of Aire and Tfh/Tfr cells in peripheral blood of MG patients by flow cytometric analysis. However, the present study has several limitations due to a limited number and further study enrolling more MG patients is needed. Based upon our results, we conclude that decreased Aire expressions are negative related to the severity of MG, and Tfh/Tfr cells are positive related to the severity of MG.

## Abbreviations

Ab: antibody
AChR: acetylcholine receptor
Aire: autoimmune regulator gene
CG: control group
GC: germinal center
GMG: generalized MG
MG: myasthenia gravis
MGFA: Myasthenia Gravis Foundation of America
OMG: ocular MG
Tfh: follicular helper T
Tfr: T follicular regulatory
RT: room temperature
